# Dysregulated neutrophilic cell death in SLE: a spotlight on ferroptosis

**DOI:** 10.1038/s41392-021-00804-z

**Published:** 2021-11-11

**Authors:** Kim Ohl, Thomas Rauen, Klaus Tenbrock

**Affiliations:** 1 Dept. of Pediatrics, RWTH Aachen University, 52074 Aachen, Germany; 2 Division of Nephrology and Clinical Immunology, RWTH Aachen University, 52074 Aachen, Germany

**Keywords:** Rheumatic diseases, Innate immune cells

In the recent issue of Nature Immunology, Li et al. present a mechanistic insight into neutrophilic cell death and its role in systemic lupus erythematosus (SLE).^1^

Several groups reported an altered neutrophil function and enhanced neutrophilic cell death in SLE patients that provoke a sustained interferon (IFN) production.^2^ In a recent issue of Nature they report that enhanced ferroptosis in neutrophils results in a breakdown of immune tolerance in SLE.^1^

**Fig. 1:**
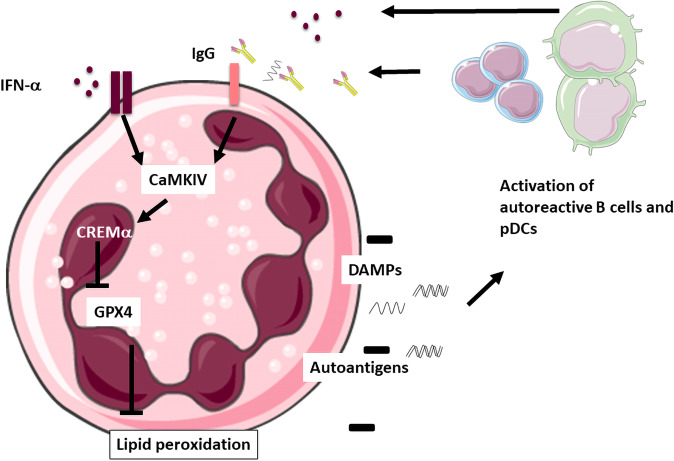
The vicious circle of neutrophil ferroptosis in SLE. Type-I IFNs and autoantibodies in SLE sera induce neutrophil ferroptosis. Neutrophilic cell death releases autoantigens and thereby activates autoreactive B cells and pDCS, which leads to further production of autoantibodies and type-I-IFNs. Images are from smart.servier.com

Ferroptosis has recently been identified as a novel type of regulated cell death that is driven by lipid peroxidation. Ferroptosis is implicated in a variety of pathological contexts such as cancer, (neuro-) degenerative diseases, but also in immune-mediated diseases such as non-alcoholic steatohepatitis (NASH), diabetes, multiple sclerosis (MS), and asthma.^3^ Nevertheless, cell-specific functions of ferroptosis remained less clear. Neutrophils are known to spontaneously undergo cell death, which is partially related to their high ROS production. ROS production is associated with increased apoptosis and delayed clearance of apoptotic cells, which are hallmark features of SLE. Neutrophil death in SLE might therefore play an important role as a source of autoantigens and of danger molecules (DAMPs) that drive IFN production and thus SLE pathogenesis. However, the exact contribution of neutrophils to pathogenesis and the underlying mechanism was uncertain. Li et al. now provide a direct mechanistic link between neutrophil death and autoimmunity. Mild to moderate neutropenia is a common finding in SLE patients. In line with this, Li et al. found reduced neutrophil counts in SLE but not in other autoimmune diseases. Compared to healthy controls, neutrophils from SLE patients exhibited a lower viability. Moreover, the viability in neutrophils from healthy controls could also be decreased by incubation with sera from SLE patients since SLE-IgG and IFN-α from SLE sera induced neutrophil cell death via ferroptosis. During cell death, neutrophils usually release DNA and dangerous molecules like high-mobility group protein (HMGB)-1 to neutrophil extracellular traps (NETs). While suppression of NETosis by the inhibitor CI-amidine treatment did not affect disease progression in a lupus mouse model, suppression of ferroptosis with Liproxstatin-1 treatment clearly mitigated disease progression. Ferroptosis is mainly regulated by Glutathione peroxidase 4 (GPX4), which uses glutathione to detoxify lipid hydroxyperoxides formed during oxidative stress. GPX4 expression was significantly reduced in SLE neutrophils but not in other immune cells, whereas reduced glutathione levels have been documented in patients with SLE before. As an in vivo proof-of-principle to study the role of neutrophil ferroptosis and GPX4 dysregulation, the authors generated myeloid-specific GPX4-haploinsufficient mice. These mice spontaneously developed a lupus-like disease, while complete ablation of GPX4 in neutrophils caused severe neutropenia but no lupus-like disease, which again provides evidence for the importance of neutrophils in SLE pathogenesis (Fig 1).

In SLE patients, reduced GPX4 expression in neutrophils was caused by IFN-α and could be induced in control neutrophils by incubation with patient SLE sera. Mechanistically, IgG-autoantibodies induced an enhanced nuclear translocation of the transcription factor cAMP response element modulator (CREM)α and its binding to the *GPX4* promoter. In prior studies, CREMα has been identified as a central factor in the T cell pathogenesis of SLE by inhibition of IL-2, induction of IL-17a expression, and enhancement in numbers of pathogenic DN T cells. Like in neutrophils, SLE-IgG induced CREMα nuclear translocation also in T cells, which resulted in increased CREMα binding to the IL-2 promotor and suppression of *IL2* gene transcription.^4^ Interestingly, SLE-IgG induced GPX4 suppression as well as lower viability was restricted to SLE neutrophils and not present in T lymphocytes. The underlying mechanism for these cell-specific effects might relate to epigenetic differences of neutrophils and T cells within accessible promotor and enhancer sequences targeted by CREMα. The higher sensitivity of neutrophils to ferroptosis might also be caused by their increased ROS exposure. Enhancing the antioxidative capacity of neutrophils including intracellular levels of glutathione might therefore reduce ferroptosis and present a possible therapeutic strategy in SLE. The Nrf2/Keap1 complex is the main antioxidative system in cells and directly involved in ferroptosis by induction of GPX4 and the cysteine/glutamate transporter SLC7A11, which was not altered in the GPX4 haploinsufficient mice. Glutamate is necessary to produce glutathione. Interestingly, older female Nrf2-KO mice spontaneously develop a lupus-like phenotype^5^ while the exact role of neutrophils in this context has not been investigated yet. However, Nrf2 is the drug target of dimethylfumarate (DMF), which has already been approved for psoriasis and MS, both diseases in which oxidative stress plays a major role. Vice versa, neutropenia is a very common side effect of treatment of MS patients with IFNα, a mechanism that could possibly be related to ferroptosis.

As a conclusion, the authors uncover a new and meaningful mechanism that drives disease initiation and progression in SLE. They thereby put a spotlight on innate immune cell abnormalities in SLE. This is of particular importance as current assumptions suggest that dysregulations of the adaptive immune system are key drivers of SLE disease. While a number of GWAS studies have identified SNPs in neutrophil-related genes as contributing risk factors in SLE, associations with GPX4 or SLC7A11 that could directly affect neutrophil ferroptosis have not been described yet. Beyond SLE it might be interesting, if neutrophil ferroptosis is also involved in other autoimmune and inflammatory diseases. The authors did not detect neutropenia in other rheumatic diseases such as RA, IBD, and AS and focused on SLE which is characterized by high type I IFN expression and chronically elevated levels of autoantibodies. High IFNα levels are also present during viral infections which often induce neutropenia. It is tempting to speculate that this is also related to the mechanism described by Li et al.

The study raises the question, if inhibition of neutrophil ferroptosis could be a treatment option in SLE. Until now it was mainly discussed with regard to cancer treatment, however, due to the sensitivity of, e.g., brain structures like the hippocampal region towards ferroptosis, side effects could be possible. Vice versa, a possible therapeutic target directly involved in ferroptosis could be the blockade of HMGB-1, which has been tried with varying success in lupus mice models. As a conclusion, ferroptosis is a very complex cell-dependent process. The underlying molecular networks and the relationship to other types of cell death require further studies.
